# Association of patient-reported outcomes and heart rate trends in heart failure: a report from the Chiron project

**DOI:** 10.1038/s41598-019-57239-4

**Published:** 2020-01-17

**Authors:** Luca Monzo, Michele Schiariti, Pietro Fedele Calvisi, Silvio Bonfiglio, Mitja Luštrek, Paolo E. Puddu

**Affiliations:** 1 “Sapienza” University of Rome, Department of Cardiovascular, Respiratory, Nephrological, Anesthesiologic and Geriatric Sciences, Rome, 00161 Italy; 2 Fimi Barco, Saronno, 21047 Italy; 3 Jožef Stefan Institute, Department of Intelligent Systems, Ljubljana, 1000 Slovenia; 4 EA 4650, Signalisation, électrophysiologie et imagerie des lésions d’ischémie reperfusion myocardique, UNICAEN, Caen, 14000 France; 5 Association for Cardiac Research, Rome, 00198 Italy

**Keywords:** Biotechnology, Cardiac device therapy

## Abstract

Patient-reported outcomes (PROs) have been previously considered “soft” end-points because of the lack of association of the reported outcome to measurable biological parameters. The present study aimed to assess whether electrocardiographic measures are associated to PROs changes. We evaluated the association between heart rate **(**HR), QRS and QT/QTc durations and PROs, classified as “good” or “bad” according to the patients’ overall feeling of health, in patients from the Chiron project. Twenty-four chronic heart failure (HF) patients were enrolled in the study (71% male, mean age 62.9 ± 9.4 years, 42% ischemic etiology, 15 NYHA class II and 9 class III) providing 1086 days of usable physiological recordings (4 hours/day). The mean HR was significantly higher in the “bad” than in the “good” PROs class (74.0 ± 6.4 bpm vs 68.0 ± 7.2 bpm; p < 0.001). Conversely, the ratio between movement and rest activities showed significantly higher values in “good” compared to “bad” PROs. We also found significantly longer QTc and QRS durations in patients with “bad” PROs compared to patients with “good” PROs. That in patients with mild to moderate HF, higher HR, wider QRS and longer QTc, as well as a reduced HR ratio between movement and rest, were associated with “bad” PROs is clinically noteworthy because the association of worse PROs with measurable variations of biological parameters may help physicians in evaluating PROs reliability itself and in their clinical decisions. Whether a timely intervention on these biological parameters may prevent adverse outcomes is important and deserves to be investigated in further studies.

## Introduction

Cardiovascular research usually focuses on “hard” end-points (i.e death and hospitalization), confining to a secondary role measures of outcome that consider patients’ symptoms and feelings. Patient-reported outcomes (PROs) are defined as “any reports coming directly from patients about how they function or feel in relation to a health condition and its therapy, without interpretation by health care providers or anyone else”^1^. PROs, that include for example symptoms, functioning, utility, adherence to therapy, health related quality of life or satisfaction with care, allow physicians to easily collect information about patients’ health status and directly measure treatment benefit beyond survival, disease and physiologic markers^1^. In heart failure (HF), PROs are raising growing interest because they are easy to collect and more reproducible than clinician-assessed symptoms, such as functional class (New York Heart Association - NYHA) or other objective clinical trial measures (i.e. ejection fraction or diastolic function)^2,3^. Moreover, the use of PROs may also outplay disparities in reported symptoms’ burden between patients and physicians, given that the latter often undervalue or even fail to recognize functional disabilities, which in turn adversely affect patient care^4^. The value of PROs in cardiovascular clinical research was also recently underscored by the European Society of Cardiology that recommended to include them in the evaluation of the efficacy of therapeutic interventions, on condition that they are assessed scientifically and rigorously^5^. However, PROs are so far considered as “soft” end-points because of their apparent lack of association with measurable biological parameters.

Resting heart rate (HR) is a strong predictor of cardiovascular mortality and morbidity in the general population^6^ and has recently gained attention as a biomarker in the management of HF patients because of its association with prognosis. In particular, in subjects with left ventricular dysfunction, an elevated HR was associated with an increased risk of all-cause mortality, cardiovascular mortality and hospitalization for worsening HF^7–10^. Interestingly, a recent report showed that the change in HR over time predicts outcome in patients with chronic HF^11^, suggesting the potential of this parameter for identification of HF patients at increased risk of rehospitalization or death.

The aim of this study was to determine whether HR and its electrocardiographic related measures (QRS and QT/QTc intervals) were associated with PROs in a series of HF patients enrolled in the European Union-Artemis funded Project Chiron^12^. We hypothesized that the higher the HR, the worse the PROs.

## Methods

### Study population

The derivation cohort was that of patients enrolled in the Chiron project^12^. The clinical characteristics and variables’ selection, primary objectives, the telemonitoring system, the protocol and the gross outcomes of this study have been previously described in detail^13^. In brief, the Chiron project conducted a clinical observational study in two countries (Italy and UK) among patients in sinus rhythm with chronic systolic HF and moderate symptoms (NYHA class II or III) with the aim of developing a comprehensive framework for personalized health management including mobile, home and hospital services^13,14^. The study was conducted in accordance with the Declaration of Helsinki and approved by site ethics committees^13^. All participants gave written informed consent to participate in the trial. Standards for reporting qualitative research (SRQR) were used^15^.

### Data collection

The Chiron patients were equipped with a wearable ECG, activity, body-temperature and sweat sensors. In addition, their blood pressure, blood oxygen saturation, weight, and ambient temperature and humidity were measured. The patients were instructed to perform daily measurements with the non-wearable devices, and used wearable devices for two hours in the morning and twice for one hour in the afternoon. The Falcon algorithm, a Time Domain Morphology and Gradient (TDMG) based algorithm, was used to extract fiducial points from the ECG signal, enabling us to compute the heart rate as well as to describe each heart beat with additional parameters such as PR interval, QRS duration and QT interval^16,17^. For QTc interval assessment, among the large number of ready correction formulas^18^ we selected two curvilinear [Bazett’s square-root^19^, and Fridericia’s cube-root formulas^20^], and one linear [Framingham’s linear regression formula^21^] equations.

The patients were also provided with a mobile application for reporting their overall feeling of health with respect to the previous day on a daily basis. They marked their overall feeling of health as one of the following options: 1) feeling much worse than yesterday; 2) feeling worse than yesterday; 3) feeling the same as yesterday; 4) feeling better than yesterday and 5) feeling much better than yesterday. The resulting data were pre-processed and analysed for relations between the objective parameters and the PROs. Since we wanted to learn the reasons for patients feeling better or worse, the occasions where the patients were feeling the same as yesterday were not interesting and we did not include them in the analysis. If each of the five distinct feelings of health corresponded to one class, the differences between them were too small to distinguish well among classes. Therefore, in order to make the difference among classes as larger as possible we decided to use only two classes corresponding to good and bad feeling of health. To identify which of the feelings should be included in the two classes, we tested nine different class definitions that included different combinations of reported feelings for different periods (i.e 1 vs 5 for one day; 1 and 2 vs 4 and 5 for two out of last three days; ect), obtaining the best differentiation between the extreme classes when patients reported to feel worse (options 1 and 2) or better (options 4 and 5) for three out of the last three to five days^13^.

### Statistical methods

Categorical data are presented as percentages, normally distributed continuous data as mean ± standard deviation (SD). Differences between groups were tested by using the Welch’s t-test for the means of two independent samples. Logistic regression analysis was used to identify the association between heart rate and PROs.

All analyses were performed using NCSS version 9 (Hintze J, Kaysville, Utah, USA: www.ncss.com). A p-value less than 0.05 was considered as statistically significant.

## Results

### Patients characteristics

A total of 38 chronic HF patients were enrolled in the study, but the data were fully analysable only in 24 (63%). Baseline characteristics of patients are presented in Table 1. They were mainly male (72%) with a mean age of 62.9 ± 9.6 years and a high prevalence of hypertension (76%). 16 patients were in NYHA class II (64%) and 9 in NYHA class III (36%) with an overall mean left ventricular ejection fraction of 34.7% ± 7.7%. At laboratory test, haemoglobin was 13.4 ± 1.6 g/L, and no significant electrolyte alterations or severe kidney impairment were found. The etiology of HF was ischemic heart disease in 10 patients (42%), idiopathic in 6 patients (25%), valvular in 2 patients (8%) and of other etiologies/cardiomyopathies in the remaining 6 patients (25%). Treatment included angiotensin receptor blockers or angiotensin converting enzyme inhibitors, diuretics, betablockers, digoxin and mineralocorticoid receptor antagonists in 80%, 88%, 84%, 16% and 48%, respectively. None of the patient was treated with amiodarone or other antiarrhythmic drugs. The enrolled 24 patients provided overall 1086 days of usable recordings (4 hours/day), including physiological data and PROs.

**Table 1:** Baseline characteristics.

Clinical characteristics	
Age, years	62.9 ± 9.6
Male, %	72
Body mass index, kg/m^2^	28.9 ± 3.9
Current smokers, %	28
Chronic obstructive pulmonary disease, %	32
Coronary artery disease, %	32
Hypertension, %	76
Diabetes mellitus, %	8
**Laboratory data**	
Haemoglobin, g/L	13.4 ± 1.6
Sodium, mmol/L	137.7 ± 4.1
Potassium, mmol/L	4.4 ± 0.4
eGFR, mL/min/1.73 m^2^	70.8 ± 20.7
Glucose, mg/dL	106.6 ± 20.9
C-reactive protein, mg/dL	2.0 (1.0; 10.5)
**Cardiac parameters**	
Left ventricular mass index, gr/m^2^	128.7 ± 28.1
Left ventricular ejection fraction, %	34.7 ± 7.7
Systolic blood pressure, mmHg	122.5 ± 14.4
Heart failure etiology, %	
- Ischemic	42
- Idiopathic	25
- Valvular	8
- Other aetiologies/cardiomyopathies	25
NYHA class, %	
- II	64
- III	36
**Treatment**	
Furosemide, %	88
ACEi or ARB, %	80
Mineralocorticoid antagonists, %	48
Betablocker, %	84
Digoxin, %	16
Devices, %	
- Implantable cardiac defibrillator	24
- Cardiac resynchronization therapy	32

ACEi – angiotensin converting enzyme inhibitor; ARB – angiotensin receptor blocker; eGFR – estimated glomerular filtration rate; NYHA – New York Heart Association.

### Heart rate and PROs

**Figure 1:**
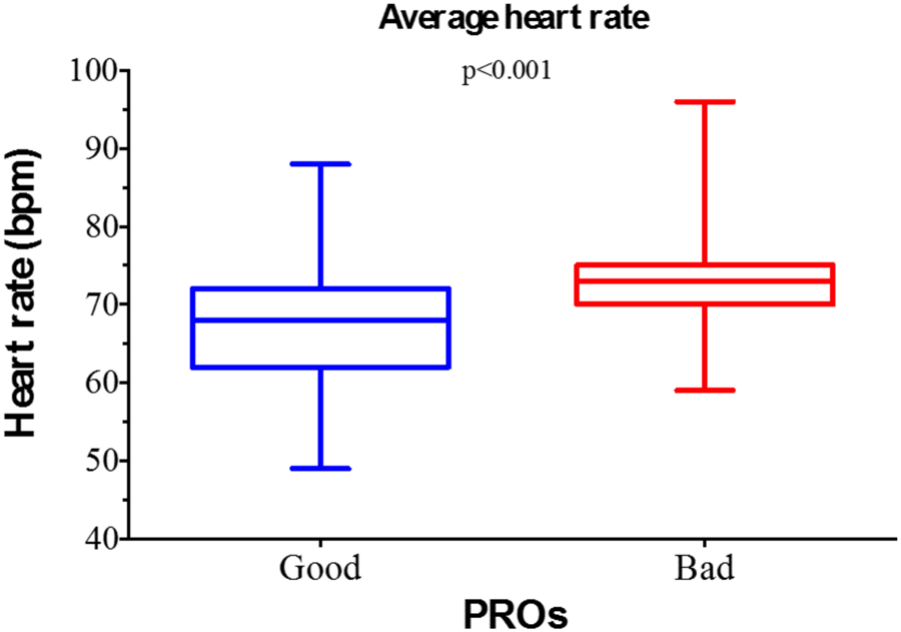
Average heart rate and the binomial (“good” and “bad”) classification of patient reported outcomes (PROs). Mid line and brackets represent mean ± SD.

**Figure 2:**
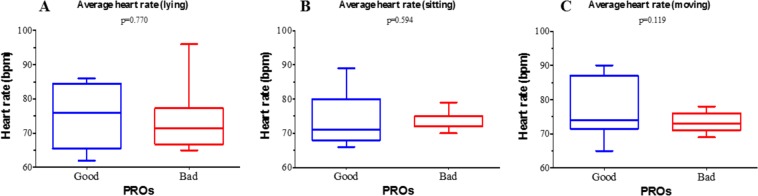
Average heart rate and the binomial (“good” and “bad”) classification of patient reported outcomes (PROs) during daily activities. (**A**) lying; (**B**) sitting; (**C**) moving. Mid line and brackets represent mean ± SD.

**Figure 3:**
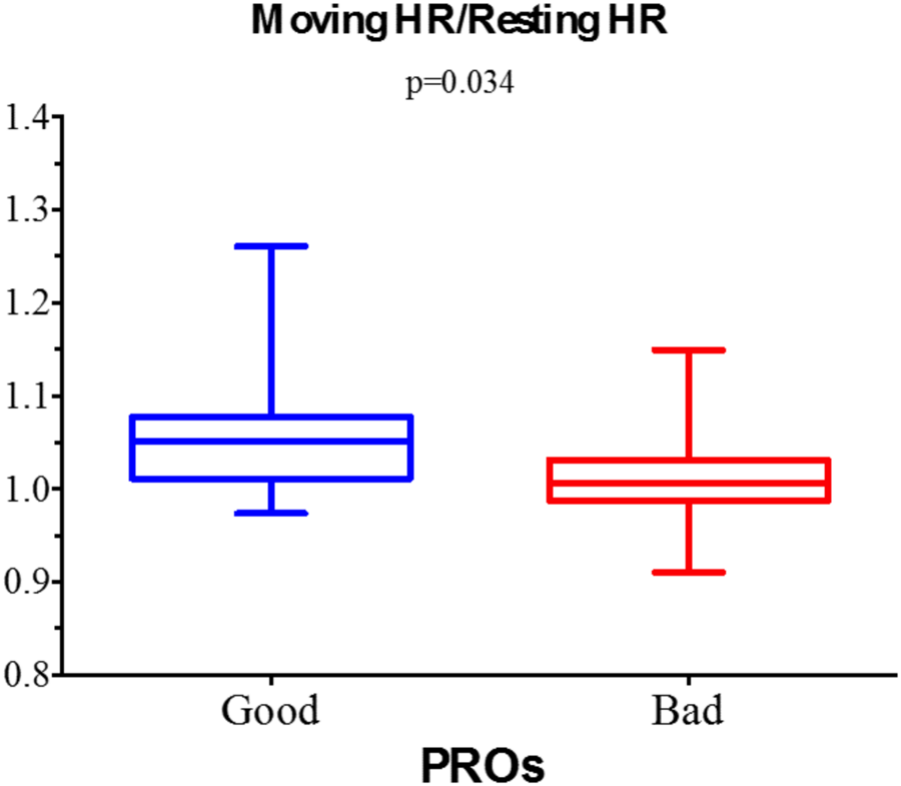
Ratio between average heart rate during movement and rest activities and the binomial (“good” and “bad”) classification of patient reported outcomes (PROs). Mid line and brackets represent mean ± SD.

The mean HR in the overall population was 69.7 ± 7.4 beats per minute (bpm). According to the binary PROs classification (bad vs good), in the “bad” class the mean HR was significantly higher than in the “good” class (74.0 ± 6.4 bpm vs 68.0 ± 7.2 bpm; p < 0.001) (Fig. 1). Conversely, we found no differences between the PRO classes either in the average HR during rest (lying: bad 74 ± 9 bpm vs good 75 ± 10.0 bpm, p = 0.770; sitting: bad 73 ± 2 bpm vs good 74 ± 8.0 bpm, p = 0.594) or during movement (bad 73.6 ± 2.7 bpm vs good 77.5 ± 7.8 bpm; p = 0.119) **(**Fig. 2**)**. Interestingly, when the ratio between the average HR during movement and rest activities was analysed, a significantly higher value was found in “good” PROs compared to “bad” PROs (bad 1.01 ± 0.04 vs good 1.05 ± 0.06; p = 0.034) **(**Fig. 3**)**.

In the logistic regression analysis HR was strongly and directly associated with PROs (regression coefficient = 0.125; p = 0.001).

### Heart rate related measures and PROs

**Figure 4:**
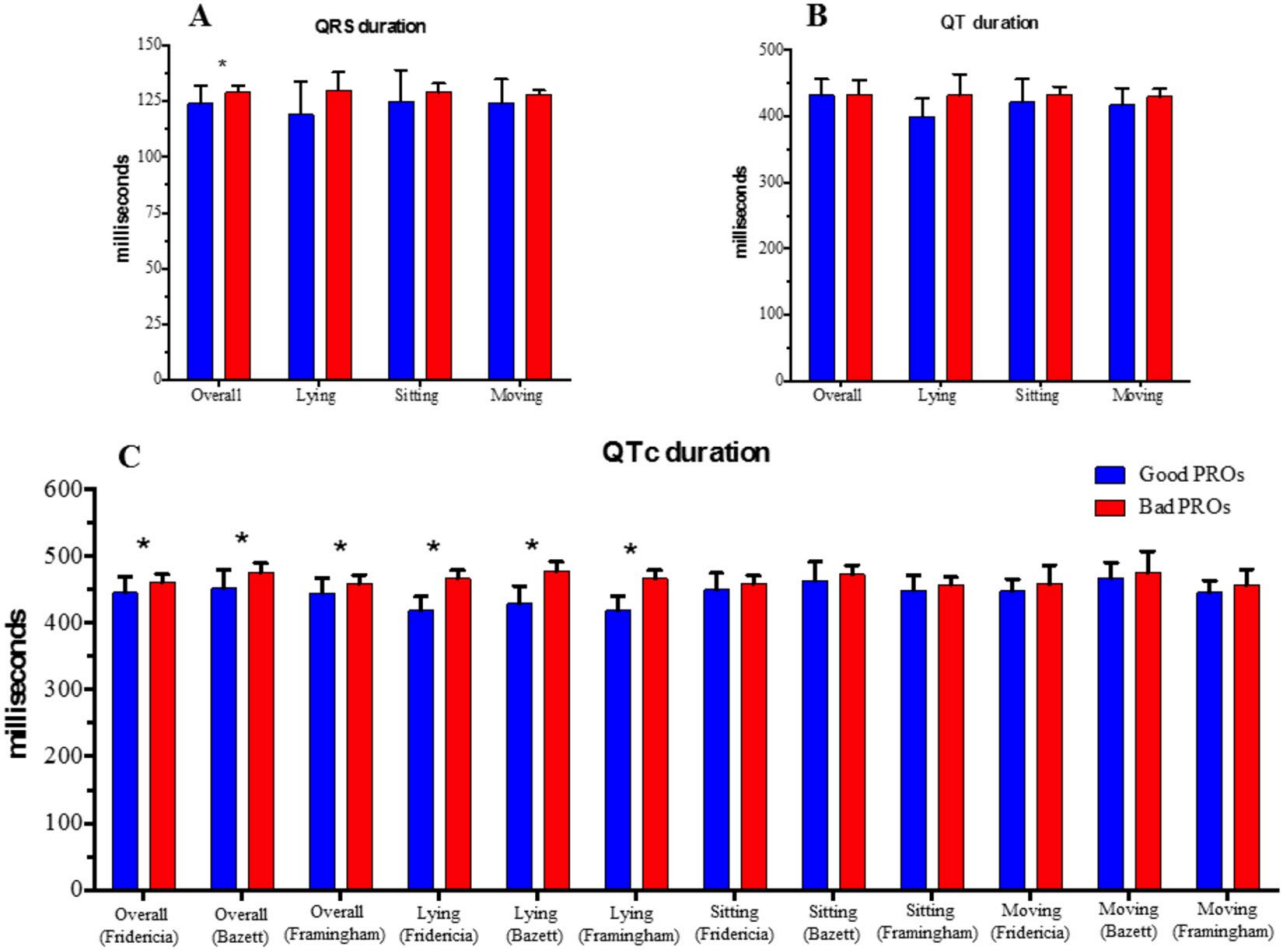
(**A**) QRS, (**B)** QT and (**C**) corrected QT (QTc) intervals duration overall and during daily activities and the binomial (“good” and “bad”) classification of patient reported outcomes (PROs). Asterisks (*) located above the bars indicate a statistically significant p-value.

We also analysed the trend of two measures closely related with HR modifications: QRS and QT/QTc intervals. We found no differences between the two PROs categories in terms of QT duration (bad 433 ± 22 msec vs good 432 ± 25 msec; p = 0.924), both overall and during movement or rest activities. Conversely, QTc average duration was significantly longer in patients with “bad” PROs compared to patients with “good” PROs, regardless of whether the Fridericia’s, Bazett’s or the Framingham formula were used. This result was mainly driven by the pronounced QTc prolongation (by all formulas) in the “bad” PROs category when patients lied down (Fig. 4 and Supplemental Table S1).

When QRS interval was analysed, patients in the “good” PROs category had a significantly narrower average QRS duration than those in the “bad” PROs category (bad 129 ± 3 msec vs good 124 ± 8 msec; p < 0.001) (Fig. 4 and Supplemental Table S1).

## Discussion

The main finding of our study is that “bad” PROs are associated with modifications of electrocardiographic parameters in patients with mild to moderate HF. In particular, higher heart rate, wider QRS and longer QTc are associated with the worst PROs.

PROs are gaining importance in clinical practice because they can provide unique information about patients’ health status, particularly in conditions such as HF where a deep understanding of patients’ symptoms and adherence to treatments, as well as their expectations, are crucial for disease management and decision making^22^. Moreover, several reports described the high reproducibility of PRO measures^2,3^, making them a reliable tool for clinic and research^23^. The main weakness of these instruments is that PROs describe something that is not directly measurable.

In our analysis higher HR was associated with worse PROs. Several observations reported that resting HR was inversely related to adverse outcomes, both in the general population and in patients with ischemic heart disease and HF^7,9,24^. This association may have multiple causes. Tachycardia reflects sympathetic overdrive, a major contributor to the progression of HF^25^. Moreover, it leads to an increased myocardial oxygen consumption and a reduction in coronary perfusion, myocardial efficiency and arterial compliance^26^. Clinical observations showed a temporal correlation between heightened HR and HF hospitalization^11^. Accordingly, increased HR may represent a marker of worsening HF providing the rationale to explain the association we have described between higher HR and “bad” PROs in our study.

When the ratio between movement and rest activities was analysed, patients who reported “good” PROs showed significantly higher values compared to when they reported “bad” PROs. One possible explanation may be related to the presence of a higher autonomic dysfunction in “bad” compared to “good” PROs patients, that in turn was reflected in a depressed heart rate variability and then in a lower heart rate ratio between movement and rest^27^. Reduced heart rate variability has been correlated to poor outcomes in a previous report^28^, and may represent another insight into the described association between electrocardiographic variables and PROs.

QT interval duration varies with several factors (i.e., age, sex, cardiac and non-cardiac disease, hemodynamic and nutritional status, drugs, etc.)^29^ and its prolongation has been associated to poor outcomes^30^. The autonomic nervous system has been shown to have a powerful influence on the QT interval^31^. HF is characterized by profound changes in autonomic function, with a markedly reduced vagal tone in favour of sympathetic overdrive^32^, that in turn can prolong the QT interval. Our results showed that QTc average duration was significantly longer in patients with “bad” PROs compared to patients with “good” PROs. One possible explanation of the QTc duration differences between patients in “good” and “bad” PRO categories may be related to the well-known heightened cardiac sympathetic drive during worsening HF^33^. Interestingly, in our study the QTc prolongation was particularly marked when patients lied down. It was previously demonstrated that prolongation of the QT interval in HF patients is more pronounced at low than at high heart rates^34^. Although the mechanisms underlying this finding are still not completely understood, the available data suggest that it may be related both to alterations in cellular processes of the cardiomyocyte and to changes in the regulation of the autonomic nervous system^35,36^.

We also demonstrated in our study an association between QRS interval prolongation and “bad” PROs. Previous observations showed as QRS interval prolongation significantly worsens outcome in HF patients^37^. QRS duration can vary widely even within the same subject and it has been reported to change maximally during HF exacerbation. Worsening HF is commonly associated with ventricular volume overload and in this setting, modifications in coronary blood flow or subendocardial ischemia induced by increased filling pressures and volume affecting conduction may determine the prolongation of the QRS complex^38^. QRS fragmentation may also contribute to this phenomenon^17^.

Our results are noteworthy since they show that PROs, classically reported as difficult to quantify because just correlated to patient’s feelings, are closely associated with measurable biological parameters, such as HR, QRS and QTc intervals. Making PROs “measurable”, the influence of “psychological dimension” (i.e. people in poor conditions could have the attitude of report their condition as constantly bad) is confined to a marginal role, enhancing in turn the value of PROs as a reliable measure of outcome in HF. Furthermore, the reported association between electrocardiographic parameters and PROs may allow the collection of information about patients’ health status and feelings by simply interpreting data obtained via remote monitoring, possibly opening a new era for the evaluation of telemonitoring outcomes. Finally, the possibility to associate PROs to variation of measurable parameters may help physicians in their clinical decision making.

Our study has several limitations that should be acknowledged. Our findings are explorative and hypothesis generating only. In fact, although Chiron study generated an enormous and refined quantity of data due to the large number of measurements and their quality, the absolute number of patients was small and does not allow any definitive conclusion about the investigated topic^13,14,16,17^. Our results were obtained in patients with chronic stable mild to moderate HF and low ejection fraction and generalizations are not possible out of this HF subgroup. The etiology of HF was not homogeneous in our cohort, and this could have influenced by various degree HR and its associated measures. Nevertheless, we did not find any significant difference between groups in HR as well as in QTc and QRS duration. This report was retrospective and observational; therefore, causality cannot be addressed. The study outcomes did not take into consideration the “psychological perspective” of the patient; namely, people in poor conditions could have the attitude of “feeling constantly bad”. Anyway, in our report we demonstrated that patients’ feelings are strictly associated to measurable biologic parameters, confining the influence of the patient’s psychological dimension on PROs to a marginal role. Finally, highly optimized and managed treatment of our cohort may have influenced measured variables to some degree. However, this should be considered a strength rather than a weakness, because it allows us to consider a real-world HF population.

Therefore, in patients with mild to moderate HF, “bad” PROs are associated with modification of electrocardiographic parameters. In particular, higher HR, wider QRS and longer QTc, as well as a reduced HR ratio between movement and rest, are associated with the worst PROs. These results appear clinically relevant because demonstrate that PROs, usually considered as a soft end-point because of its nature difficult to define and influenced by external factors such as the physiological perspective of patients, are associated with measurable variations of biological parameters that may help physicians not only in evaluating PROs reliability itself but also to timely intervene, or even prevent, worsening of HF patient’s health status. In particular, whether a timely intervention on the cited biological parameters may prevent adverse outcomes is of particular clinical interest and deserves to be investigated in further studies. Indeed, this hypothesis was specifically considered when the HeartMan Project was started based on the idea that various interventions including physical exercise, psychological support and dietary recommendations might be instrumental in lowering HR and preventing adverse PROs^39^ thus ameliorating health-related quality of life in patients living with HF^40^.

### Ethics approval and consent to participate

This study was approved in Italy by the ethics committee of Policlinico Umberto I Hospital of Rome, reference 39/13 of January 17, 2013 and in United Kingdom by the Lothian NHS Board, South East Scotland Research Ethics Committee 02, reference 12/SS/0101 of July 26, 2012.

## Supplementary information

Supplementary Table 1.

## Data Availability

Data might be requested to Mitja Lustrek at Jožef Stefan Institute, Department of Intelligent Systems, 1000 Ljubljana, Slovenija who acted as Principal Investigator of the Center where the data of the Chiron Project were deposited.
