# Supplementary material for: Association of patient-reported outcomes and heart rate trends in heart failure: a report from the Chiron project
Source: Sci Rep. 2020 Jan 17;10:576. doi: 10.1038/s41598-019-57239-4 (PMC6969136; doi:10.1038/s41598-019-57239-4)
Supplement: Supplementary file 1 — Supplementary Table 1. [file 41598_2019_57239_MOESM1_ESM.docx]

**Association of patient-reported outcomes and heart rate trends in heart failure: a report from the Chiron project**

Luca MONZO ^(a)^, Michele SCHIARITI^(a)^, Pietro Fedele CALVISI^(a)^, Silvio BONFIGLIO^(b)^, Mitja LUŠTREK^(c)^, Paolo E. PUDDU^(a,d,e)^,

on behalf of the Chiron and HeartMan research projects

1. “Sapienza” University of Rome, Department of Cardiovascular, Respiratory, Nephrological, Anesthesiologic and Geriatric Sciences, 00161 Rome, Italy
2. Fimi Barco, 21047 Saronno, Italy
3. Jožef Stefan Institute, Department of Intelligent Systems, 1000 Ljubljana, Slovenija
4. EA 4650, Signalisation, électrophysiologie et imagerie des lésions d’ischémie reperfusion myocardique, UNICAEN, 14000 Caen, France
5. Association for Cardiac Research, 00198 Rome, Italy

**E-mail addresses:** [**luca.monzo@uniroma1.it**](mailto:luca.monzo@uniroma1.it.com)**;** [**michele.schiariti@uniroma1.it**](mailto:michele.schiariti@uniroma1.it)**;** [**pf.calvisi@gmail.com**](mailto:pf.calvisi@gmail.com)**;** [**silvio.bonfiglio@barco.com**](mailto:silvio.bonfiglio@barco.com)**;** [**mitja.lustrek@ijs.si**](mailto:mitja.lustrek@ijs.si)**;** [**puddu.pe@gmail.com**](mailto:puddu.pe@gmail.com%20it)

**Short title: Patient-reported outcomes and HR in CHF**

**Words:** Title 17; Abstract: 249; Text: 2717.

**Number of figures:** 4; **Number of tables:** 1

**Supplementary material:** Table: 1; Figures: 0.

**References:** 40 (1130 words)

Author for correspondence and reprints: P.E. Puddu, MD, PhD, FESC, FACC, Sapienza University of Rome, Department of Cardiovascular, Respiratory, Nephrological, Anesthesiologic and Geriatric Sciences, Viale del Policlinico, 155, Roma 00161, Italy. Tel. +39.06.49972659; Fax. +39.06.4453891; e-mail: [puddu.pe@gmail.com](mailto:puddu.pe@gmail.com)

**Supplementary Table 1.** QRS, QT and QTc average duration overall and during lying, sitting and moving according to the binomial (“good” and “bad”) classification of PROs.

|  | **Good** | **Bad** | **p-value** |
| --- | --- | --- | --- |
| **QRS interval** | | | |
| QRS average duration overall - msec | 124 ± 8 | 129 ± 3 | <0.001 |
| QRS average duration lying - msec | 119 ± 15 | 130 ± 8 | 0.189 |
| QRS average duration sitting - msec | 125 ± 14 | 129 ± 4 | 0.225 |
| QRS average duration moving - msec | 124 ± 11 | 128 ± 2 | 0.183 |
|  | | | |
| **QT interval** | | | |
| QT average duration overall - msec | 432 ± 25 | 433 ± 22 | 0.924 |
| QT average duration lying - msec | 400 ± 27 | 432 ± 32 | 0.071 |
| QT average duration sitting - msec | 422 ± 35 | 433 ± 12 | 0.239 |
| QT average duration moving - msec | 418 ± 25 | 430 ± 12 | 0.129 |
|  | | | |
| **Corrected QT interval** | | | |
| QTc average duration overall (Fridericia) - msec | 446 ± 24 | 461 ± 12 | <0.001 |
| QTc average duration overall (Bazett) - msec | 452 ± 28 | 476 ± 14 | <0.001 |
| QTc average duration overall (Framingham) - msec | 445 ± 23 | 460 ± 12 | <0.001 |
| QTc average duration lying (Fridericia) - msec | 419 ± 21 | 467 ± 12 | 0.004 |
| QTc average duration lying (Bazett) - msec | 429 ± 27 | 478 ± 14 | 0.011 |
| QTc average duration lying (Framingham) - msec | 419 ± 21 | 465 ± 11 | 0.004 |
| QTc average duration sitting (Fridericia) - msec | 450 ± 25 | 459 ± 12 | 0.233 |
| QTc average duration sitting (Bazett) - msec | 464 ± 28 | 473 ± 14 | 0.269 |
| QTc average duration sitting (Framingham) - msec | 449 ± 23 | 458 ± 11 | 0.210 |
| QTc average duration moving (Fridericia) - msec | 448 ± 18 | 459 ± 28 | 0.224 |
| QTc average duration moving (Bazett) - msec | 468 ± 23 | 476 ± 32 | 0.453 |
| QTc average duration moving (Framingham) - msec | 446 ± 18 | 457 ± 24 | 0.184 |

List of abbreviations: msec, milliseconds; PROs, patient reported outcomes; QTc, corrected QT interval.
